# Epidemiological Characteristics of Severe Fever with Thrombocytopenia Syndrome from 2010 to 2019 in Mainland China

**DOI:** 10.3390/ijerph18063092

**Published:** 2021-03-17

**Authors:** Xiaoxia Huang, Jiandong Li, Aqian Li, Shiwen Wang, Dexin Li

**Affiliations:** 1 NHC Key Laboratory for Medical Viruses and Viral Diseases, National Institute for Viral Disease Control and Prevention, Chinese Center for Disease Control and Prevention, Beijing 102206, China; huangxx@ivdc.chinacdc.cn (X.H.); ldong121@126.com (J.L.); liaq@ivdc.chinacdc.cn (A.L.); 2 Center for Biosafety Mega-Science, Chinese Academy of Sciences, Wuhan 430071, China

**Keywords:** severe fever with thrombocytopenia syndrome, epidemiological characteristics, surveillance

## Abstract

Severe fever with thrombocytopenia syndrome (SFTS) is an emerging infectious disease and that is a severe threat to public health considering its high fatality and person-to-person transmission. In order to obtain an updated and deep understanding of the epidemiological characteristics of SFTS in mainland China, we used Pearson’s chi-squared test to compare the fatality rate and demographic characteristics in different groups. Data were analyzed in R3.6.1 (R Development Core Team 2018), while the visualization was performed in ArcGIS 10 (ESRI, Redlands, CA, USA), and the statistical significance was set at *p* < 0.05. A total of 13,824 SFTS cases involving 8899 lab-confirmed cases and 4925 probable cases were reported and included in the epidemiological analysis. Our study found that the number of SFTS cases showed an increasing trend with a small decrease in the past three years. The laboratory-confirmed rate was about 64.4%, which varied between different years and areas. Although most cases (99.3%) were distributed in 7 provinces (Henan, Shandong, Anhui, Hubei, Liaoning, Zhejiang, and Jiangsu), the regional distribution of SFTS gradually expanded from 5 provinces in 2010 to 25 provinces by 2019, especially at the town level. The SFTS cases were mainly sporadic. A total of 96.5% occurred from April to October, and 93.3% of cases were concentrated in middle-aged and elderly people (40–84 years old). Farmers were the main high-risk population. Female cases were slightly more than male cases; however, there were differences between different provinces. The mortality rate showed an increasing trend with age. Overall, the SFTS cases were mainly middle-aged and elderly farmers that sporadically distributed throughout seven provinces with a spatially expanding trend. The laboratory-confirmed rate varied in different years and provinces, which implied that the diagnosis and report criteria for SFTS should be further updated and unified in order to get a better understanding of its epidemiological characteristics and provide scientific data for SFTS control.

## 1. Introduction

Severe fever with thrombocytopenia syndrome (SFTS), caused by the SFTS virus (SFTSV), which was named the *Dabie bandavirus* of the *Bandavirus* genus of the Phenuiviridae family by the International Committee on Taxonomy of Viruses, is an emerging viral infectious disease with a high fatality rate [1,2]. Fever, thrombocytopenia, and leukocytopenia are its main clinical manifestations, which vary in severity from mild to multiple organ disorders and even death. An early study showed that the initial fatality rate reached 30% [1]. SFTS is mainly transmitted by ticks, where *Haemaphysalis longicornis* is its main vector [3,4]. SFTSV is detected in ticks collected from domestic animals (e.g., sheep, dog, and cattle), vegetation, and patients [3,5,6]. SFTSV is most likely circulated between ticks and some animals and is transmitted to humans via tick bites [3]. Furthermore, SFTS can be transmitted through directly contacting patients’ blood, which is also an important transmission route, especially in the cluster of SFTS cases observed in several high endemic areas [7,8,9].

SFTS was first identified in China in 2009; then, SFTS-like virus (heartland virus) infections were reported in the United States in 2012; in 2013, Japan and South Korea confirmed their first SFTS cases; Vietnam has also reported SFTSV infections in recent years [1,10,11,12]. In China, SFTS monitoring has been conducted nationwide since 2010 [13], where SFTS case data is required to be reported to the National Notifiable Disease Report System (NNDRS). A previous study [14] focused on lab-confirmed cases between 2010 and 2017 showed that SFTS was highly sporadically distributed in seven provinces with a 6% fatality rate, where farmers aged 50–74 years constituted a high-risk population.

Although SFTS has been recognized for more than 10 years, it is still an important public health issue in China because of its high fatality rate and human-to-human transmission. We conducted this study while considering the lack of updated epidemiologic characteristics of SFTS in mainland China. In this study, we analyzed the SFTS epidemiological characteristics between 2010 and 2019, which aimed (1) to give a comprehensive and updated epidemiological overview of SFTS in mainland China and (2) to provide further basic data for scientific prevention and control against SFTS.

## 2. Materials and Methods

### 2.1. Data Collection and Management

In this study, SFTS data from 2010 to 2019 were obtained from the NNDRS, which is a disease-monitoring network operating from the county to provincial levels in China. SFTS case information can be exported for further analysis through this system with permission. The SFTS cases analyzed involved lab-confirmed cases and probable cases. The definition of lab-confirmed cases was mentioned in our previous study [15]. Probable cases are those that have more clinical manifestation and/or laboratory detection results than a suspected case [15], as diagnosed by clinicians, but without a specific pathogen detection result. The case records that were retrieved for statistical analysis mainly included the current living address, age, gender, occupational, onset date, case type (lab-confirmed case or probable case), case survival, and other indicators. The data extraction and use in this study were all anonymized to protect patient privacy and confidentiality.

### 2.2. Data Analysis

The geographical distribution of SFTS cases was analyzed according to the “current living address.” R3.6.1 (R Development Core Team 2018) was used to conduct the statistical analysis. Pearson’s chi-squared test was conducted to compare the fatality rates and demographic characteristics in different groups. Statistical significance was set at *p* < 0.05. ArcGIS 10 software (ESRI, Redlands, CA, USA) was used to demonstrate the spatial distribution of the SFTS cases at the provincial level.

## 3. Results

### 3.1. Overview of SFTS in Mainland China

**Figure 1:**
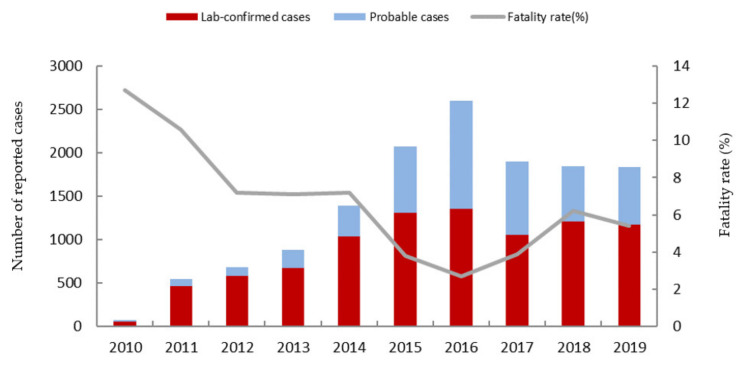
The number of severe fever with thrombocytopenia syndrome (SFTS) cases and the fatality rate from 2010 to 2019.

From 2010 to 2019, a total of 13,824 SFTS cases involving 8899 lab-confirmed cases and 4925 probable cases were reported in mainland China, with 713 deaths. The average annual fatality rate was 5.2% nationwide. The number of SFTS cases showed an increasing trend between 2010 (71 cases) and 2016 (2600 cases), which then decreased to 1838 cases in 2019. However, the annual fatality rate showed an opposite changing trend, which decreased from 2010 (12.7%) to 2016 (2.7%) and then increased to 6.2% in 2018, with a slight decrease in 2019 (5.4%). Figure 1 shows the number of SFTS cases and the fatality rate changing trend from 2010 to 2019.

From 2010 to 2019, the total laboratory confirmation rate was 64.4% (8899/13,824), which showed differences between different years. The highest was observed in 2012 (84.9%) and the lowest in 2016 (52.1%). Among the seven provinces with the highest SFTS incidence, the total laboratory confirmation rates were the highest in Zhejiang and Jiangsu, which were 100% (459/459) and 95.2% (296/311), respectively, then followed by Shandong (77.2%, 3082/3990), Liaoning (68.6%, 486/708), Henan (60.1%, 2482/4132), Hubei (58.2%, 1080/1856), and Anhui (42.7%, 971/2274).

### 3.2. Analysis of the SFTS Epidemiological Characteristics from 2010 to 2019

#### 3.2.1. SFTS Regional Distribution

**Figure 2:**
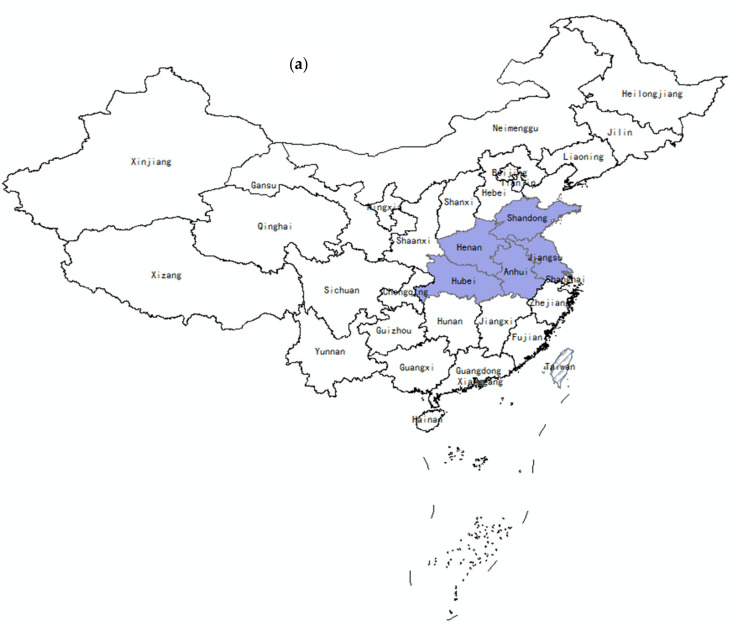

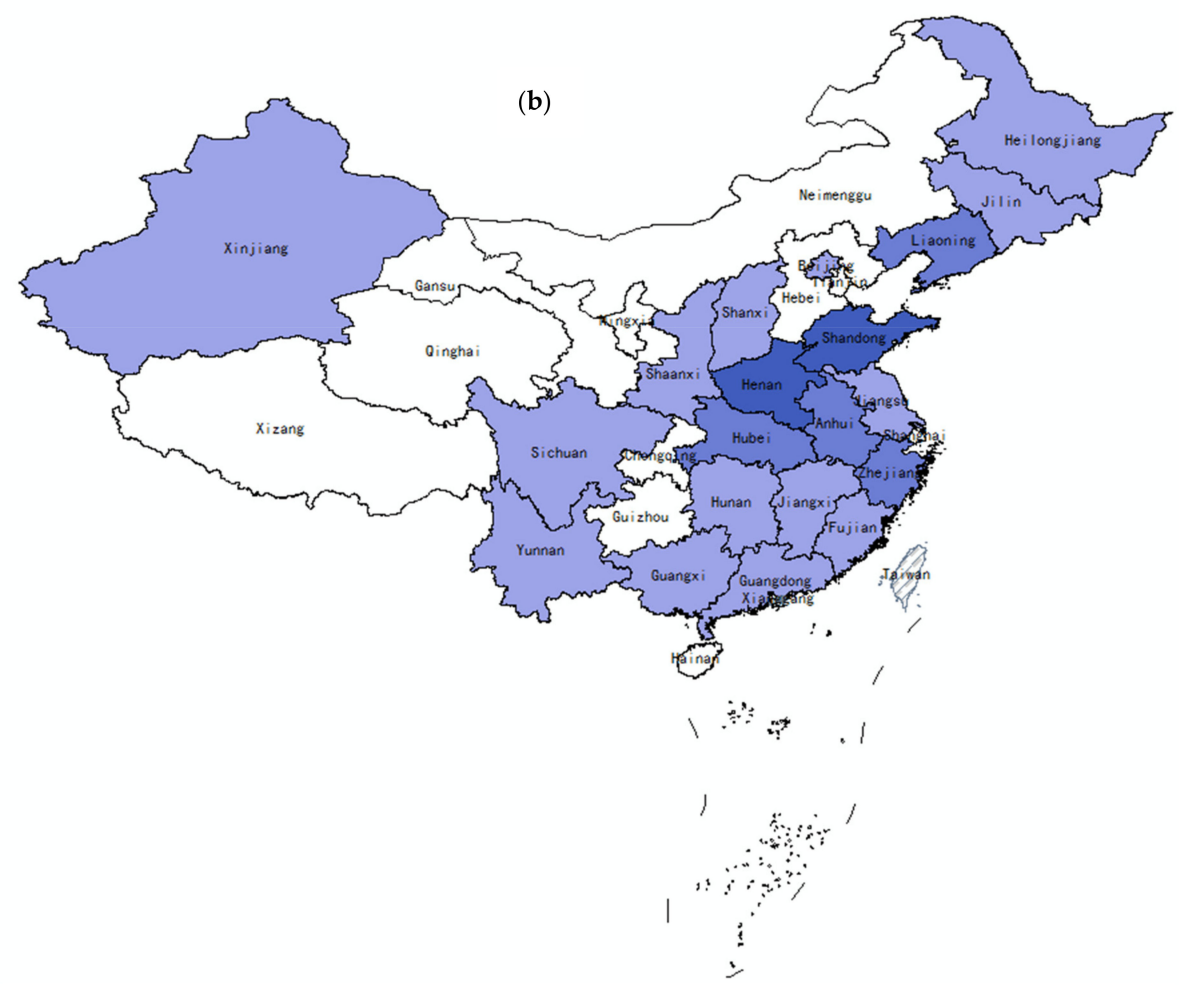

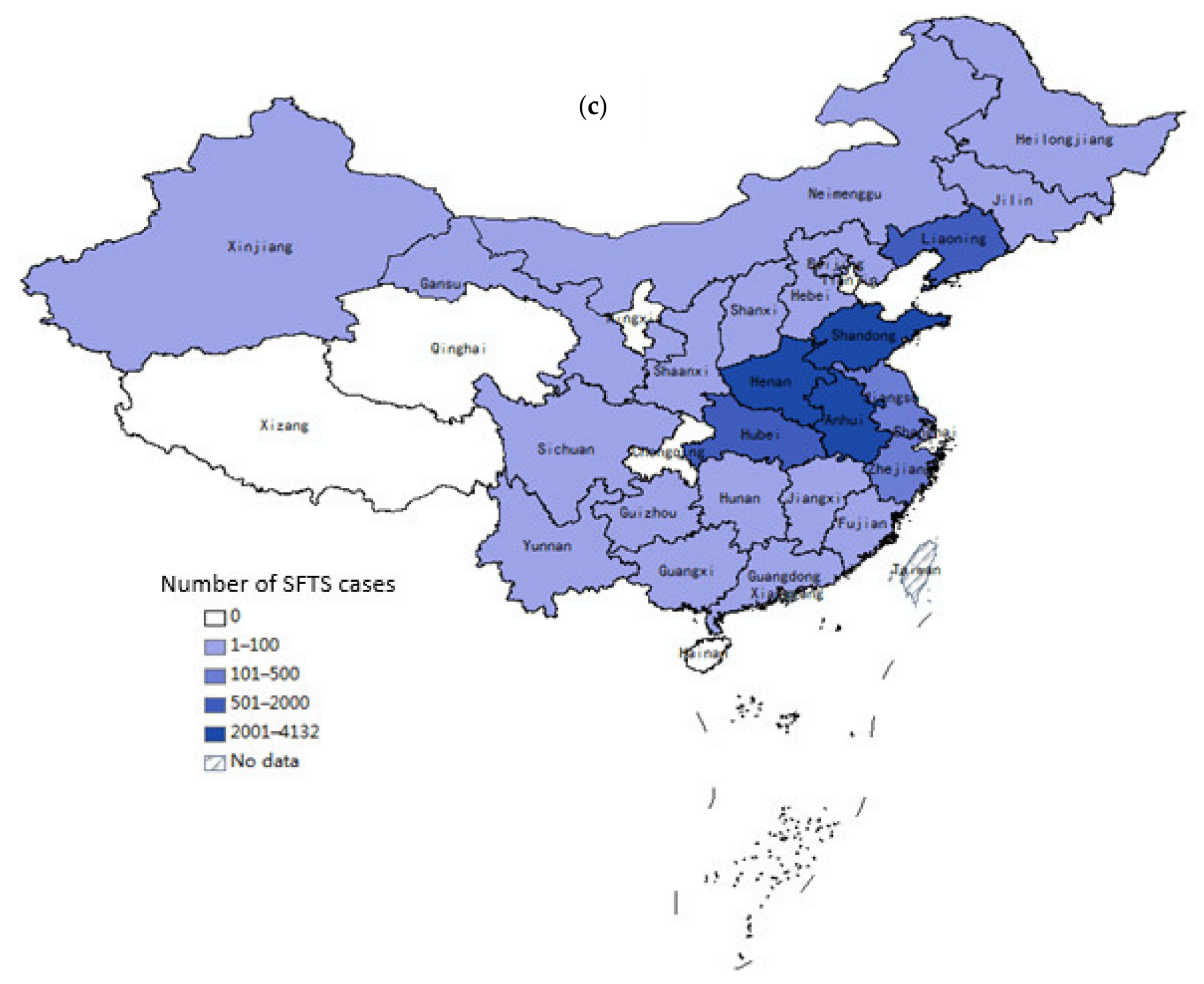
Regional distribution of SFTS cases in mainland China: (**a**) SFTS distribution in 2010, (**b**) the accumulative SFTS distribution from 2010 to 2014, and (**c**) the accumulative SFTS distribution from 2010 to 2019.

From 2010 to 2019, the accumulative number of provinces that reported SFTS cases increased from 5 to 25. However, the SFTS cases were still mainly distributed in seven provinces located in central, eastern, and northeastern China, consisting of Henan (4132 cases), Shandong (3990 cases), Anhui (2274 cases), Hubei (1856 cases), Liaoning (708 cases), Zhejiang (459 cases), and Jiangsu (311 cases), which accounted for 99.3% (13,730/13,824) of the total reported cases in mainland China (Figure 2).

At the town level, 42 towns reported 71 cases in the first monitoring year (2010); the total number of towns where SFTS cases have been reported increased to 2433 by 2019. From 2010 to 2019, 1162 out of 2433 (47.8%) towns only reported in one year, 971 (39.9%) reported in 2–5 years, 295 (12.1%) towns reported in 6–9 years, and only 5 towns (0.2%) reported SFTS cases every year; most (60.1%, 3831/6377) towns reported only one case in one year, which varied between 53.1% (494/931) in 2016 and 72.4% (254/351) in 2011, and only 2.4% (150/6377) towns reported ≥10 cases in one year, which varied from 0 in 2010 to 5.1% (39/767) in 2015. The town with the highest annual SFTS cases (42 cases) was in Henan province in 2015.

#### 3.2.2. SFTS Temporal Distribution

**Figure 3:**
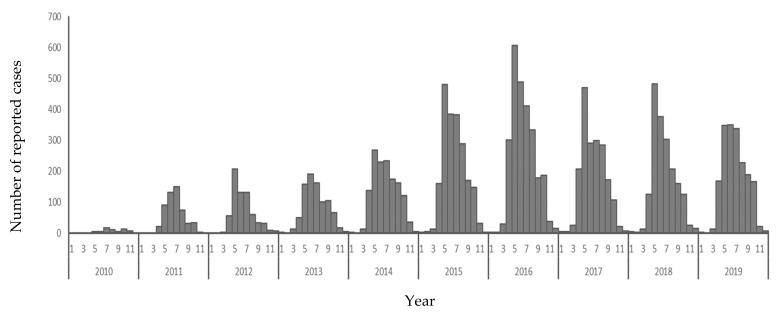
Monthly SFTS distribution in mainland China from 2010 to 2019.

According to the overall incidence from 2010 to 2019, the SFTS cases occurred every month, but were mainly distributed between April and October (96.5%, 13,344/13,824) and peaked in May (Figure 3). The cases reported from May to July accounted for 58.8% (8128/13,824) of the total SFTS cases in mainland China and 60.3%, 59%, 55%, 63.5%, 47%, 63%, and 59.2% in Henan, Shandong, Anhui, Hubei, Liaoning, Zhejiang, and Jiangsu, respectively.

#### 3.2.3. SFTS Population Distribution

During the observed period, the youngest case was a 2-month-old male (probable case) and the oldest case was a 100-year-old female (lab-confirmed case). The 40–84-years-old age group accounted for 93.3% (12,895/13,824) of the total SFTS cases, ranging from 88.7% (63/71) in 2010 to 94.5% (518/548) in 2011.

The overall male-to-female ratio of SFTS cases was 0.88:1, which was similar in all the observed years, except in 2010 (1.45:1) and 2011 (1.06:1). The total gender distribution was different in seven highly endemic areas (*χ*^2^ = 128.344, *p* = 0.000). The number of male cases was more than the number of female cases in Jiangsu (1.30:1), Liaoning (1.19:1), and Shandong (1.02:1) and was less in Henan (0.67:1), Hubei (0.88:1), Zhejiang (0.93:1), and Anhui (0.93:1).

The SFTS occupational analysis showed that 86.5% (11,958/13,824) of SFTS cases were farmers, followed by household workers and unemployed (6.7%) and retirees (2.5%).

### 3.3. SFTS Death Analysis

**Figure 4:**
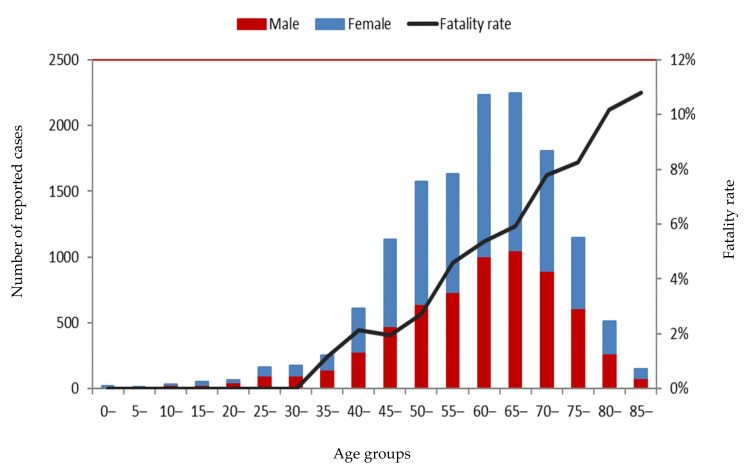
Population distribution of SFTS cases from 2010 to 2019.

A total of 713 deaths were observed in 10 provinces between 2010 and 2019. Among the above mentioned seven highly endemic provinces, Zhejiang (11.5%) and Shandong (10%) had the highest fatality rates, followed by Jiangsu (5.8%), Hubei (4.5%), Anhui (3.5%), Liaoning (3.2%), and Henan (1.3%). There was a statistical difference (*χ*^2^ = 372.815, *p* = 0.000) regarding the fatality rates in these provinces. The deaths were only observed in persons aged ≥37 years old. When the SFTS data was observed in 5-year age intervals, the fatality rate showed an increasing trend with age, where the highest fatality rate was observed in the ≥85-years-old age group (Figure 4). The male-to-female ratio of death cases was 1.16:1, which was different from that of survival cases (*χ*^2^ = 14.373, *p* = 0.000).

## 4. Discussion

From 2009 to 2010, the SFTS virus was first discovered by Chinese experts through using molecular biology, virus isolation, serology, and other technical methods [2]. The disease caused by the SFTS virus infection was named SFTS [13], which is an acute infectious disease with a high fatality rate and is mainly transmitted by a tick bite [1]. Based on the early knowledge of SFTS, China began to conduct surveillance, report, and engage in prevention and control in mainland China. SFTS is required to be reported in terms of the requirements of a notifiable class B infectious disease. In this study, based on all SFTS case data retrieved from the NNDRS from 2010 to 2019, we carried out a systematic analysis of the epidemiological characteristics at the national, provincial, and/or town levels.

Our results showed that more than 99% of SFTS cases were still reported from seven high-risk provinces located in central, eastern, and northeastern China, namely, Henan, Shandong, Anhui, Hubei, Liaoning, Zhejiang, and Jiangsu. Although most cases were concentrated in these seven provinces, the SFTS cases were mainly sporadic, where the areas with SFTS cases showed a gradual expansion trend, which may be related to the widespread distribution of *Haemaphysalis longicornis*, the main vector of the SFTS virus in China, as well as the improvement in the SFTS diagnosis procedure. SFTS was mainly transmitted by tick bites, and its temporal distribution was closely related to some elements such as the activity time of ticks [16] and the fieldwork of the population. SFTS cases occurred mainly in April to October, peaking during May and July, which was similar to results found in a previous study [17]. SFTS cases were mainly middle-aged and elderly farmers, who were considered to have a greater chance of contacting the ticks. Considering the wide distribution of ticks and the increasing number of areas with reported SFTS cases, the regions current without SFTS case reports should also be paid more attention to, and prevention and control publicity, as well as diagnosis training, should be conducted to improve the awareness of prevention and diagnosis among the population to achieve early prevention and treatment.

SFTS is an emerging hemorrhagic fever, its fatality rate (5.2%) is much higher than that of other forms of viral hemorrhagic fevers in China, such as hemorrhagic fever with renal syndrome and dengue fever. It is still an important health issue in China. The changing trend in yearly SFTS fatalities showed a downward trend and then increased, which was almost opposite to the yearly reported number of cases. There were differences in the case fatality rate (CFR) between key provinces, where Zhejiang had the highest CFR, followed by Shandong, and Henan had the lowest CFR. The SFTS fatality rate is considered to be related to the case age, viral load of infection, course of the disease, and local diagnosis and treatment levels [18,19]. Further study should be focused on the different fatality rates in different high-risk areas. Age-specific mortality showed an increasing trend with age, which was similar to that of 2011–2014 [17].

The SFTS laboratory-confirmed rate was about 64.4%, which varied between different years or areas. Since the first discovery of SFTS, it has been in China for many years and the medical and public health get increasingly more understanding of this disease; to this end, our study suggested that it is necessary to further unify the diagnostic and report criteria for SFTS, which are of great significance for understanding its epidemic characteristics.

To interpret the results of the study, a few limitations should be noted. First, SFTS national surveillance was first conducted in late 2010; therefore, only a few cases were reported in 2010. Second, increasingly more cases were identified with the promotion of detection technology and the deepening understanding of this disease.

## 5. Conclusions

Our study systematically analyzed the epidemiological characteristics of all SFTS cases reported in mainland China. The SFTS cases were mainly middle-aged and elderly farmers that were mainly found in seven provinces, though we should note that the regional distribution had an expanding trend. Therefore, it was suggested that SFTS should be emphasized nationwide and the diagnosis and reporting procedures should be updated based on further detailed research, which can lead to a better understanding of the epidemiological characteristics and provide scientific data for SFTS control.

## Data Availability

All available data were presented in this study.
